# A Case of Closure of a Vesico-Vaginal Fistula at a Second Operation

**Published:** 1865-04

**Authors:** J. R. Lothrop


ART. III.—*A Case of Closure of a Vesico-Vaginal Fistula at a second operation.* BY J. R. LOTHROP, M. D.

In this case the fistula followed a first labor. It occurred, as do most others which follow childbirth, after a long labor, lasting from Tuesday to the following Saturday. During this time the woman was attended by a midwife and a physician in succession, and had been subjected to the administration of both ergot and chloroform. From the patient's statement it seems probable that the head remained low down in the pelvis more than forty-eight hours, and that the urine was allowed to accumulate largely in the bladder. Saturday evening Dr. Wyckoff first saw the patient, and immediately removed the child by instruments. It had the appearance of having been some time dead, perhaps attributable in part to the ergot. Apprehensive that a fistula might be caused by the long continued pressure, Dr. W. visited the patient until all danger of its occurrence seemed over, and then ceased attendance.

Three weeks and three days after the confinement, the patient states that she was wakened in the night by a great and sudden flow of urine, so great as to pass through the bedding to the floor. After this the retentive power of the bladder was lost. Dr. Wyckoff was then called, and found that an opening between the bladder and vagina existed. Ascertaining by examination that the opening was small, he kept Sims' catheter constantly in the bladder, and after a time made use of caustic applications to the fissure; hoping thereby to produce a closure. The size of the fistula, and the almost complete prevention of escape of urine by the vagina, which followed the use of the catheter, taken in connection with the natural tendency to contraction and union which is commonly, in such cases, observed, encouraged the expectation of cure by this plan of treatment. But this plan failing an operation was decided upon. I had seen the patient with Dr. Wyckoff several times while the above treatment was pursued. At his request and with his assistance, together with that of Drs. Brown, Miner and Boardman, I made an operation for the closure of the fistula on the 24th of January last. The first operation failing, a second was made on the 18th of February following, which proved successful. The delivery took place on the 15th of October, 1864; the fistula was established three weeks and three days, or twenty-four days subsequently; and the operations were made as above stated.

Cause.—From the above statement, it will be apparent that a period longer than usual elapsed before the opening into the bladder occurred. Commonly, sloughing following protracted labor perforates the vesico-vaginal septum in less than two weeks, often in a few days. This perforation though hastened by, is not dependent upon distension of the bladder by accumulated urine. But a case may occur in which the slough only partially destroys the septum, leaving a thin wall between the bladder and vagina. In such a case, the thin partition would be likely to give way under the pressure of a large amount of urine in the bladder, or if violent efforts were made to evacuate it. The case then would be somewhat like rupture of the bladder. Though the weakening of the vesico-vaginal septum was caused by sloughing following pressure in a protracted labor, still a fistula would not occur if the bladder was frequently emptied.

The statements of the patient warrant the inference that, in this case something similar took place. She stated that for a few days before she lost the power of retaining the urine, she had great difficulty in emptying the bladder, and that several times she was unable by great efforts to force any urine from the bladder, for one or two days. The great and sudden flow of urine which took place in the night, happened after retention, as she stated, for two or three days. The existence of granulating surfaces at several points in the vagina was quite certain indication of a destruction of, at least, the mucous membrane of the vagina at those points, and that restoration was taking place. The same process was probably going at the point where the fistula was found, but the destruction having been greater, there was not tissue enough to prevent rupture under the pressure of a large amount of urine. These considerations seem necessary to explain the occurrence of the fistulous opening at so long a period as twenty-four days after delivery.

Description.—The fistula was transverse in direction, from a half to three-fourths of an inch in length, situated about an inch and a half from the urethral orifice, and to the right of the median line. It was therefore low down, near the neck of the bladder, and as regards size and situation favorably circumstanced for operation. The edges moreover were but slightly hardened, and the fistula being of a size to admit the end of the fore-finger, did not in any great degree give to it the sensation of a cartilaginous ring.

Operations.—The patient, under the influence of ether, was placed in the position for lithotomy. The first step in the operation, viz: the paring of the edges, was mainly done, by the aid of a small hook, a pair of long scissors, bent on the flat in a short curve, and a short glass speculum, through which the fissure was easily reached. The speculum was then withdrawn, and a steel staff introduced into the bladder, to bring it down, in order that, with the aid of retraction, the paring might be completed, and the stitches, four in number, inserted. The particular form of suture employed was that known as the "clamp suture," the crossbars being of lead, half round and perforated; the sutures of silk. The stitches were made in the usual way; passed as deeply as was possible without perforating the mucous coat of the bladder, and secured by a shot at each end of the suture. Short needles, slightly curved, and a pair of Sims' needle forceps, were employed in making the stitches. Sims' catheter was then introduced into the bladder to be kept in for a few days. No urine escaped by the fistula for several days, but at about the fourth day it was apparent that the closure was not perfect. An examination was made at about the seventh day. It was then found that the stitches had cut their way out by ulceration, on one side, and that the operation had failed; the fistula being, if any change had occurred, larger than before its closure was attempted.

In three or four weeks a second operation was made. The preparatory process in this trial was a repetition of the first, except that a knife was employed instead of the scissors, and the paring was wholly down through the speculum. It was then removed, and again the staff and retractors employed. The "clamp suture" was not made use of, but instead silver wire was employed for sutures. Four stitches were put in, and the wire was secured by twisting. The wires were cut, leaving ends about three-fourths of an inch in length. Some attempt was made to bend over the ends, that they might do as little injury as possible. The catheter was worn as before. About the twelfth day no escape of urine having occurred the stitches were removed. Nearly two months have elapsed since the second operation; the closure remains complete, and the bladder has resumed its proper functions.

Remarks.—The short glass speculum employed was, one of large size cut off for the purpose, with a sloping end as in the longer ones; the longest side being perhaps two inches in length. This served a very good purpose in the paring of the edges of the fistula, affording a good light and easy access to the fissure. Indeed the whole process of closure might as readily have been effected by using it.

The position of the patient cannot always be predetermined. Something will depend upon the position of the fistula. If the position chosen is that for lithotomy, ether or chloroform can be much more easily given, and the patient is under easier management. The position upon the hands and knees has this advantage, that the flow of urine, which is rather troublesome in the opposite position, is prevented during the operation, and perhaps it affords a better exploration of the vaginal cavity. But if an anæsthetic is made use of, there is great difficulty in maintaining the patient in the position, and besides as more or less propping is demanded, pressure upon the abdomen can hardly be avoided. It therefore interferes with that dependence of the abdomen which is quite an important feature of the position. The use of an anæsthetic in this as in many other operations, seems certainly of much importance, not only to prevent pain, but to suspend voluntary movements and resistance, which in this case are troublesome.

The failure of the first operation cannot fairly be set down to the form of suture used, though we can easily conceive that thin parts pinched between the cross-bars may be, in a measure, strangulated, and hence more liable to slough. The use of silk instead of silver for sutures, was a departure from the original plan of the "clamp suture," and had perhaps some agency in causing the failure. Nothing should be set down against the "clamp suture" when it is not fully employed. The desire to produce exact apposition, in the closure of a vesico-vaginal fistula, may lead to considerable pinching of the included tissues when the wires are secured, and thus aid in defeating the aim of the operator. Of the "clamp suture," some one remarked: "on the whole, I am convinced that it is the best form of suture that we yet have." But recent practice indicates that it has ceased to be regarded as the best form of suture. It is complicated and consumes much time in adjustment, and is not followed by better results than the simpler and more easily applied form of interrupted suture, when silver wire is used.

After all that has been written about improvements in the methods of closing vesico-vaginal fistulæ, and after all the complicated contrivances, which were intended to render, and have been thought to have rendered success more certain; if we except the use of silver wire instead of silk for sutures, there has been no great gain over the simpler method adopted and recommended by Dr. Hayward. The essential points, as he maintained, are; to pare the edges well, to obtain a good surface for adhesion; to include abundant tissue within the sutures, by deep stitches; to allow the sutures to remain a considerable time, if of silk till they slough out; and to keep a catheter constantly in the bladder for a number of days. The use of the staff was an important feature also in the operation, for by it the bladder could be easily brought down. In all essential points this is the method employed by many successful operators, as for instance, Mr. I. Baker Brown of London. The staff may be replaced by a hook or forceps. The use of silver wire instead of silk appears to be a gain in method. Dr. Hayward allowed his silk sutures to work their way out by sloughing, which they will do generally in a few days. Silver wire will undoubtedly remain longer and cause less sloughing. He thought the catheter might be removed after the third day, but still employed at regular intervals to remove the urine, thereby avoiding all danger of breaking up newly formed adhesions.

In the case above related the catheter was worn constantly for about three weeks, a longer time than was perhaps necessary, but it caused no trouble or inconvenience, and it was thought best to continue its use. The patient lay upon her back mostly, and it was not found necessary to give opiates, as very little pain followed. Some operators are careful to keep the patient after the operation, upon the side, with the knees drawn up.

It is not many years since Mr. Earl said, "these cases present great obstacles, and are certainly the most difficult that occur in surgery." In one case he performed "upwards of thirty operations" before he succeeded. As the difficulties are largely mechanical, we may not be surprised that modern ingenuity has overcome them in a great degree. It is about twenty-six years since the first successful operation was made in this country by Dr. Hayward. He operated twenty times on nine patients. The results were, in three cases complete success; in five partial; in two failure. Comparing this with the cases of Mr. Brown of London, we find the number of successful cases much greater. In the London Surgical Home fifty-five cases were operated upon by him. The results were, as far as they had been determined, in forty-three cases perfect cure; in one partial; in five failure; in two death. Of the forty-three successful cases, in twenty-four one operation only was made; in eight two; in five three; in six more than three.

Thus in the earlier periods of the operation, three cases in nine succeeded, while in recent operations we have forty-three in fifty-five successful. We find that in sixty cases thirty-six or more than half were cured by a single operation.

